# **Effect of Tamoxifen and Lithium on Treatment of Acute Mania Symptoms in Children and Adolescents**

**Published:** 2016

**Authors:** Elham Fallah, Sorror Arman, Mostafa Najafi, Bahar Shayegh

**Affiliations:** 1 Child Psychiatry Department, Behavioral Sciences Research Center, Isfahan University of Medical Sciences, Isfahan, Iran.; 2 Child Psychiatrist, Isfahan university of medical sciences, Isfahan, Iran; 3 Department of Psychology, Islamic Azad University, Sharekord Branch, Sharekord, Iran

**Keywords:** Lithium, Mania, Protein Kinase C, Tamoxifen, Children

## Abstract

**Objective:**

Many studies have supported the role of protein kinase C (PKC) inhibitors in the physiopathology and treatment of bipolar disorder in adults. Tamoxifen is one of the drugs with the effect of PKC inhibition. This study aimed to determine the effect of tamoxifen on the rate of improvement mania symptoms in the sample of children and adolescents with acute mania.

**Materials & Methods:**

In this randomized, placebo-controlled clinical trial study, registered in www.irct.ir with the code of IRCT201410126418N3, overall 44 patients with bipolar disorder with acute manic episode were randomly assigned into treatment and control groups. The serum levels of lithium and tamoxifen among the participants in the treatment groups were 0.8 -1.1 mg and 20-40 mg per day respectively. Serum level of lithium among participants in the control group was similar. The main comparisons were made based on the Young Mania Rating Scale (YMRS) and Children Depression Inventory (CDI) scores of the participants at baseline and at the end of each study week. The pharmacological side effects of serum level of lithium were examined weekly. Analysis of Covariance(ANCOVA) test was used for the statistical analysis.

**Results:**

There was no difference in the baseline score of YMRS and CDI in the treatment and control groups while a statistical significant difference (P < 0.05) in these scores was found between and within the groups.

**Conclusion:**

The addition of tamoxifen to lithium causes a significant difference in reducing the symptoms of mania and depression in the treatment group compared to the control group.

## Introduction

The identification of protein kinase C (PKC) has recently been introduced as a promising therapeutic aim. Protein kinase C is a large family of isoenzymes typically found in brain and plays a crucial role in settings before and after synapse called neurotransmitter. PKC facilitates the release of dopamine (1). Regulation of longterm changes in gene and neuronal plasticity is another important role of PKC (2,3). The pathway of PKC signals regulates the morphology of dendritic branches (4). 

PKC activity and change in platelets of patients with mood disorder is in response to protein, which increases the percentage of response in patients with mania, besides it is reduced in patients treated by lithium (5).

In rodents, PKC stimulation caused by cocaine stimulus makes the excitation of manic symptoms. On the other hand, the inhibition of PKC activity by lithium and valproate results in the reversal of the manic symptoms (6, 7). Amphetamines create manic-like behaviors in rodents through the effect on PKC and phosphorylation of GAP.43 (playing a role in the release of dopamine neurotransmitter) (8-10). The use of lithium, valproate and tamoxifen inhibit the increase of amphetamine-induced activity (11).

Since the late 1990s, some drugs have been made based on the activity of PKC inhibition, while the mandatory characters to test such drugs in humans include: having the ability for PKC inhibition in areas of the brain involved in the mood disorder, evaluating this ability in more than an animal model, passing easily from Blood- Brain Barrier (BBB) and tolerating the drug absorption. 

The best acceptable candidate with PKC inhibitory feature that meets the above characters is Tamoxifen (12). Tamoxifen is a synthetic anti-estrogen drug used for the treatment of breast cancer (12) and the prevention of osteoporosis (13). Due to the lack of metabolic and neurological side effects, compared to antipsychotic drugs, it is a good alternative in the treatment of acute mania (14). Drugs recognized to have interact with tamoxifen are 2D6 and P150 drugs such as paroxetine, sertraline and duloxetine (15) and sodium valproate, Plus phenytoin and carbamazepine (16). Early and before maturity onset of a bipolar disorder, usually becomes chronic and appears without specific periods and with high rate of mixed mania (17). The disease is comorbid in onset, worsened by other group of disorders (18) and at an increasing rate of comorbidity (19). Therefore, an appropriate, timely, effective and low risk treatment is essential for bipolar disorder in children. However, there are a few drug-controlled studies in this field (20). Shortterm studies on clinical samples in America showed that early-onset of Bipolar I disorder is associated with slow response rate to treatment, persistent mood swings, high recurrence rate, high risk of attempted suicide and severe psychosocial disorders (21). Therefore, an aggressive treatment of mania and prevention of manic episodes can minimize the risk of manic-depressive disorders (22). However, intolerance to some side effects such as metabolic disorders and overweight are other problems that require a special attention and cause some limitations to use of these drugs (23). Prevention of recurrence plays an important role in the treatment of bipolar disorder. The primary objective of recent advanced treatment methods is control of the acute phase of the disorder before advancing into the chronic stage. This objective can be achieved by the use of available antimania, antipsychotic and anticonvulsants drugs. With the exception of lithium, these three categories of drugs have no priority one over the other (24). Furthermore, no interaction has been reported between tamoxifen and lithium (25). Anti-manic drugs such as lithium and valproate have influence over PKC (26). 

There are some reports concerning the effect of tamoxifen in treating bipolar disorder in adults. However, there was no controlled study to evaluate the effect of tamoxifen in children and adolescents with bipolar disorder (8, 27).

Studies on children who suffered from desmoids fibromatosis gynecomastia (27) and low-grade brain gliomas (28) and who were on tamoxifen therapy up two years reported no serious complications. This has paramount importance because similar drugs with appropriate doses that are used in adults can work for children and adolescents (29). Thus, prompt treatment of manic episodes and preventing the occurrence of chronic course of the disorder and possible side effects of some anti-manic drugs such as valporates and second generation, antipsychotics starting from childhood is vital.

This study was aimed at measuring the effect of tamoxifen as an adjuvant of lithium in reducing the rate of symptoms of mania in children as well as adolescents with mood disorder.

## Materials & Methods

This randomized, double-blinded clinical trial registered in www.irct.ir with the code of IRCT201410126418N3, was conducted on nine to 20 yr old patients with the diagnosis of acute mania hospitalized at Al-Zahra University Hospital-Psychiatric Ward, Isfahan, Iran. 

The total sample size was 44 patients of which 22 were assigned in the treatment group (receiving tamoxifen + lithium) and the remaining 22 in the control group (receiving placebo+ lithium).

Written consent was obtained from the guardian or parent of each patient after explaining the methods and aims of the performance. In addition, the therapeutic method was verbally explained the study participants based on their level of understanding and developmental stage. 

This study has been approved by ethical committee of Isfahan University of Medical Sciences with the code of 393501.

The inclusion criteria for the study subjects were the age range of 9-20 yr old, the diagnosis of bipolar disorder based on K-SADS interview, the Young Mania Rating Scale (YMRS) score of above 20, cutting or not taking any medication that can affect the mind for at least 2 weeks prior to the study, independence from any narcotic and psychotropic drugs for one month before the study had begun, IQ score of greater than 70 and no major nervous and physical diseases such as epilepsy. 

Lack of desire to continue in the study and the creation of intolerable side effects were the exclusion criteria.

**Instruments for data collection:**

1. The questionnaire of demographic characteristics including age and education level
2. K-SADS Semi-structured interview Diagnosis of the bipolar disorder, schizophrenia, and its type and episode was performed prior to treatment using K-SADS interview. The reliability and validity of the Persian version for assessment and diagnosis of psychiatric disorders in children have been reported as accepted (30).
3. The questionnaire of Young Mania Rating Score (YMRS) is a measure filled by a physician after interviewing a patient, parents and if possible, employees in the psychiatric ward. This questionnaire is made up of 11 items which are scored in 4 levels and for each level a definition for that item has been provided. The scores ranged from zero to 6. The reliability of the test has been reported in the range of 0.41 to 0.85 and its validity is 0.89 compared to the Patterson Mania and 0.88 compared to the Total Mania Rating Tests. The cut-off point for diagnosing manic disorder is a score of above 20. This test enables to differentiate bipolar disorder from Attention Deficit-Hyperactivity Disorder (ADHD) and is to some extent sensitive to treatment with mood stabilizers (31).
4. Maria Kovacs’s Child Depression Inventory (CDI): is like Beck’s Depression inventory and is leveled as zero, 1 and 2. The range of scores is from zero to 54 and the cut-off and diagnosis point of depressed patients is a score of more than 20. In order to measure the validity, Concurrent validity measure of CDI scale by teachers has shown a score of 0.193, which was significant (32).
5. Table of side effects (33) Somnolence during day, morning Somnolence, constipation, dizziness, fatigue, restlessness, tremor, increasing appetite, loss of appetite, blurred vision, diarrhea, dry mouth, nervousness.

Based on randomized blocks, patients were divided into two equal groups that placebo+ lithium was administered to one group, while tamoxifen + lithium were administered to the other group. The study subjects were given 300 mg dose of lithium on the first day. Based on the patient’s tolerance level and weekly monitoring for its serum level, the dose was increased to 0.8-1.1 mg/l. Again, tamoxifen was started with daily standard-dose for children, about 20 mg, and increased to a maximum of 40 mg/d (33).

Patients of developed insomnia or agitation were managed with lorazepam at a starting dose of 1 mg to 4 mg for the first four days of hospitalization and then, the dose for the following days was reduced to 2 mg. Placebo, made up of sucrose, was started alone once a day in the control group receiving lithium and it was increased to twice a day. The questionnaires for YMRS, CDI and drug side effects were filled before intervention and in the first, second and third weeks. 

Response to treatment was evaluated at 50% reduction in the rate of YMRS (30), using SPSS version 18 (Chicago, IL, USA). Age and sex were controlled for the ANCOVA and the changes in the CDI and YMRS scores in the first, second and third weeks of study were compared between the intervention and control groups.

## Results

**Fig 1:**
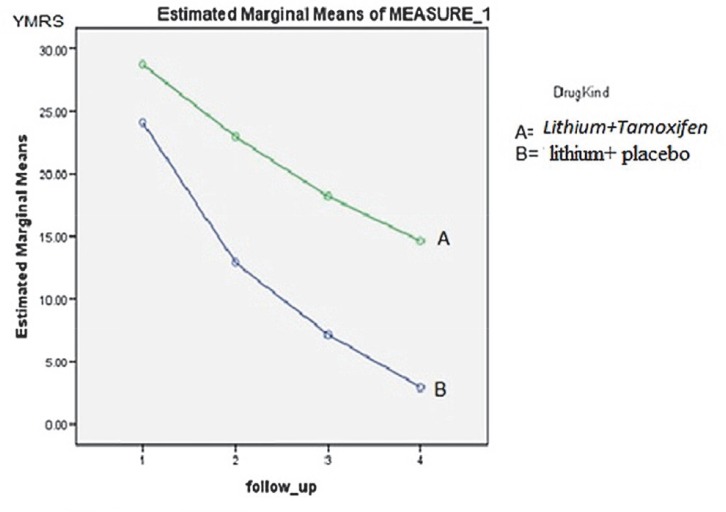
Covariates appearing in the model are evaluated at the following values: Age=14.5±1.9 (yr) With regard to Table 3, there was no statistically significant difference (F3 and 39 = 0.602; P= 0.67) between follow-up periods, while a significant difference (F1 and 41= 26.6; P = 0.001) was identified between the intervention and control groups.

Figure 1 shows the trend of estimated marginal means Since the late 1990s, some drugs have been made based on the activity of PKC inhibition, while the mandatory characters to test such drugs in humans include: having the ability for PKC inhibition in areas of the brain involved in the mood disorder, evaluating this ability in more than an animal model, passing easily from Blood- Brain Barrier (BBB) and tolerating the drug absorption. 

The best acceptable candidate with PKC inhibitory feature that meets the above characters is Tamoxifen (12). Tamoxifen is a synthetic anti-estrogen drug used for the treatment of breast cancer (12) and the prevention of osteoporosis (13). Due to the lack of metabolic and neurological side effects, compared to antipsychotic drugs, it is a good alternative in the treatment of acute mania (14). Drugs recognized to have interact with tamoxifen are 2D6 and P150 drugs such as paroxetine, sertraline and duloxetine (15) and sodium valproate, Plus phenytoin and carbamazepine (16). Early and before maturity onset of a bipolar disorder, usually becomes chronic and appears without specific periods and with high rate of mixed mania (17). The disease is comorbid in onset, worsened by other group of disorders (18) and at an increasing rate of comorbidity (19). Therefore, an appropriate, timely, effective and low risk treatment is essential for bipolar disorder in children. However, there are a few drug-controlled studies in this field (20). Shortterm studies on clinical samples in America showed that early-onset of Bipolar I disorder is associated with slow response rate to treatment, persistent mood swings, high recurrence rate, high risk of attempted suicide and severe psychosocial disorders (21). Therefore, an aggressive treatment of mania and prevention of manic episodes can minimize the risk of manic-depressive disorders (22). However, intolerance to some side effects such as metabolic disorders and overweight are other problems that require a special attention and cause some limitations to use of these drugs (23). Prevention of recurrence plays an important role in the treatment of bipolar disorder. The primary objective of recent advanced treatment methods is control of the acute phase of the disorder before advancing into the chronic stage. This objective can be achieved by the use of available antimania, antipsychotic and anticonvulsants drugs. With the exception of lithium, these three categories of drugs have no priority one over the other (24). Furthermore, no interaction has been reported between tamoxifen and lithium (25). Anti-manic drugs such as lithium and valproate have influence over PKC (26). 

There are some reports concerning the effect of tamoxifen in treating bipolar disorder in adults. However, there was no controlled study to evaluate the effect of tamoxifen in children and adolescents with bipolar disorder (8, 27).

Studies on children who suffered from desmoids fibromatosis gynecomastia (27) and low-grade brain gliomas (28) and who were on tamoxifen therapy up two years reported no serious complications. This has paramount importance because similar drugs with appropriate doses that are used in adults can work for children and adolescents (29). Thus, prompt treatment of manic episodes and preventing the occurrence of chronic course of the disorder and possible side effects of some anti-manic drugs such as valporates and second generation, antipsychotics starting from childhood is vital.

This study was aimed at measuring the effect of tamoxifen as an adjuvant of lithium in reducing the rate of symptoms of mania in children as well as adolescents with mood disorder.

**Fig 2:**
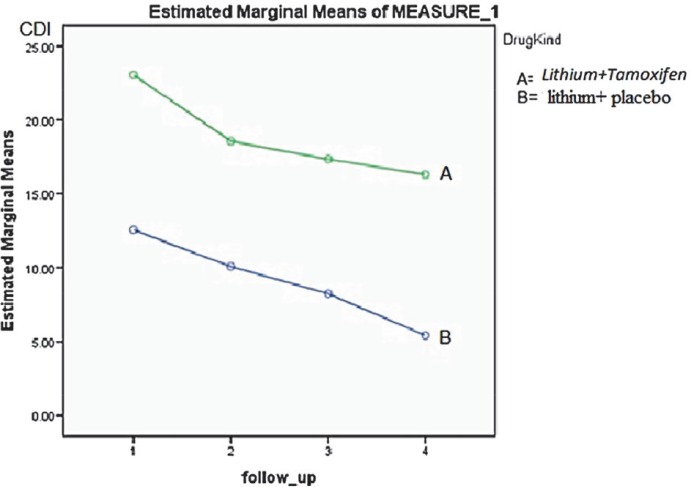
Covariates appearing in the model are evaluated at the following values: Age=14.5±1.9 (yr)

## Discussion

In this clinical trial, the addition of tamoxifen to lithium resulted in an increased rate of clinical improvement, especially in the first week of treatment. A significant decrease in YMRS and slight decline in CDI scores were observed.

Treatment with 40 mg tamoxifen per day is well tolerated in children and there were no serious side effects and complications observed except dry mouth. The efficiency of tamoxifen in reducing manic symptoms in this study supports the hypothesis that PKC plays a great role in the treatment of mania. The effects of tamoxifen on central nervous system are complex and appear to be a serotonergic system antagonist. However, it has no stimulatory effects in cholinergic and adrenergic systems (31, 32).

In another study, maniac symptoms were created in mice by injecting amphetamine in which the symptoms were controlled through injecting tamoxifen for 14 days and it seemed that tamoxifen had controlled dysfunction in hippocampus and frontal area (33). The use of tamoxifen in Kun-Albright syndrome in children has been approved (34). In a study on 7 boys with precocious puberty treated by tamoxifen with the dose of 10-20 mg for 2 years, no side effect was reported (35).

Another study conducted on 59 patients aged between 10-18 yr with desmoid fibromatosis treated with a high dose of tamoxifen for two years helped to control the underlying diseases and reported no specific side effects (36). In a study on 38 adolescents with gynecomastia of puberty period at the age group of 14.6 years treated by tamoxifen with the dose of 40 mg for 3 and 9 months, no specific side effects were reported; however, the underlying disease was controlled (37). In a study on 14 children under 14 year-old with the diagnosis of lowgrade brain gliomas treated by tamoxifen with the dose of 20 mg for one year, it operated well without specific side complication in the control of gliomas (38). In 5 studies on adults, the effect of tamoxifen in the decrease of symptoms of mania was is consistent with our results; however, no study was found on the effect of tamoxifen on the mood of children. In a single blind study on 7 subjects in the age group of 18-65 yr old (5 females and 2 males), tamoxifen could have a good performance in the treatment of acute mania (39). Tamoxifen only with the dose of 20-40 mg was tolerated for 15 days well (39). 

The anti-mania effect of tamoxifen on 51 women was compared with that of medroxy progesterone acetate for 4 weeks and the anti-mania effect of tamoxifen with the dose of 80 mg per day was not attributed to its antiestrogenic effects, and it was resulted from the blocks of receptors of protein kinase C (40). In another study for 35.4±7.8 years old participanrs (41) and in the study on the age group of 18-65 yr old (42), as monotherapy with a high dose (140 mg per day (40) and 80 mg per day (41), tamoxifen was used to treat patients with bipolar disorder by current or mixed mania episode for three weeks. They also evaluated the effect of tamoxifen on other symptoms such as depression and psychosis. Both above-mentioned studies showed the high ability of tamoxifen as a potent inhibitor of PKC to improve the symptoms of mania in which the psychotic symptoms was controlled too (41, 42). However, in Hamilton Rating Scale for Depression (HRSD), there was no significant change in comparison with placebo (41, 42). The combined treatment of lithium with tamoxifen led to a clear mood stability and even the control of symptoms of psychosis in bipolar disorder (43). In the present study, maximum recovery time and reduction in YMRS score were observed in the first week. In addition, in the mixed stage of mania cases, CDI score was dropped by controlling the underlying disease. In none of the participants in treatment group, depression was not developed following the use of tamoxifen and this is consistent with the findings of studies on adult patients with bipolar disorder or current or mixed mania episode (40, 43).

The development of depression symptoms were usually reported following the long-term use of tamoxifen and in patients who had an innate potential to depression (44). 

In a study on 2943 patients with breast cancer treated by prolonged monotherapy with tamoxifen with the dose of 80 mg, the incidence of New-onset depression in tamoxifen recipients was not significant (45). However, it seems that a short-term treatment (three weeks) with the low-dose of tamoxifen in patients with bipolar disorder not only causes the induction of depression but also controls the symptoms of depression in the mixed cases through the control of acute symptoms of the disease (24).

No subject left the study due to the side effects of the drug and only dry mouth in the tamoxifen group was more common than that in the placebo group. The present study is pioneer in the investigation of the effect of tamoxifen, which showed the effect on acute phase of the disorder is consistent with the similar results of the disorder in adults (20, 22, 38, 40).

However, the low number of studied samples, lack of examining associated disorders as comorbidity with bipolar disorder including psychosis as well as the lack of investigation drug effect alone (monotherapy with tamoxifen) are among the limitations of the study. It is noted that tamoxifen has been effective as monotherapy (41-42) and in improving the prognosis of psychosis among adults (43).

**In conclusion, **the addition of tamoxifen to lithium showed a significant difference between the treatment and control groups in reducing the symptoms of mania and depression.

**Table 1:** Within group comparison of CDI and YMRS scores at baseline and at the end of first, second and third weeks of follow up the study participants with bipolar disorder treated by tamoxifen or placebo

**Variable**	**Tamoxifen -Lithium**	**Lithium** ** -** **Placebo**	**P.Value**
**Mean of YMRS** * ** SD-(Base)** **Mean of CDI** # ** SD-(Base)**	24.5±5.4 13±6.5	7±28 11±23	0.094 <0.001
**Mean of YMRS SD-(First Week)** **Mean of CDI SD-(First Week)**	6±13 5±15	7±23 18.5±10.5	<0.001 <0.002
**Mean of YMRS SD-(Second Week)** **Mean of CDI SD-(Second Week)**	6.4±7 4±8	8±18 9±17	<0.0001 <0.0001
**Mean of YMRS SD-(Third Week)** **Mean of CDI SD-(Third Week)**	3.5±3 4±6	9±14 7±16	<0.0001 <0.0001

* Young Mania Rating Scale (YMRS),

# Children Depression Inventory

**Table 2:** Results of covariance with replicated test score with the result of mania score in the study population controlling age and sex

**Variables**	**F value**	**DF**	**P.Value**
**Follow up duration**	2.42	3.37	0.081
**Intervention Group (Lithium+Tamoxifen/Lithium+Placebo)**	3.42	1.39	0.001
**The interaction of follow up duration and intervention groups**	3.25	3.37	0.032

**Table 3:** Results of the analysis of covariance (ANCOVA) with replication with the outcome of depression score in the population studied controlling age and sex

**Variables**	**F value**	**DF**	**P. value**
**Follow up duration**	0.602	3.39	0.617
**Intervention Group (Lithium+Tamoxifen/ Lithium+Placebo)**	26.6	1.41	0.001
**The interaction of follow up duration and intervention groups**	0.592	3.39	0.624

**Table 4:** Comparison of side effects in the two studied groups

**Side Effect**	**Lithium+Tamoxifen**	**Lithium+Placebo**	**P.value**
**Drowsiness**	0.22	2.22	0.488
**Vertigo**	0.22	2.22	0.488
**Shivering**	1.22	0.424	0.254
**Overeating**	0.22	1.22	1
**Anorexia**	0.22	2.22	0.488
**Blurred vision**	1.22	2.22	1
**Fatigue**	0.22	1.22	1
**Agitation**	1.22	0.22	1
**Xerostomia**	7.22	0.22	0.009
**Anxiety**	0.22	2.22	0.488
**Diarrhea**	0.22	0.22	1
**Constipation**	0.22	0.22	1
**Low body movement**	0.22	0.22	1

## References

[1] Manji HK, Lenox RH (1999). Ziskind- Somerfeld Research Award Protein Kinase C signaling in the brain: Molecular Transduction of mood stabilization in the treatment of manic-depressive illness. Biol Psychiatry.

[2] Calabrese B, Halpain S (2005). Essential role for the PKC target MARCKS in maintaining dendritic spine morphology. Neuron.

[3] Craske ML, Fiveaz M, Batad NN, Meyer T (2005). Spines and neurite branches function as geometric attractors that enhance protein Kinase C action. J Cell Biol.

[4] Friedman E, Hoan Yan W, Lewinson D, Connell TA, Singh H (1993). Altered Platelet Protein Kinase C activity in bipolar affective disorder, manic episode. Biol Psychiatry.

[5] Wang Hy, Freidman E (1996). Enhanced Protein Kinase C activity and translation in bipolar affective disorder brain. Biol Psychiatry.

[6] Einat H, Yuan P, Szaho ST, Dogra S, Manji HK (2007). Protein Kinase C inhibition by tamoxifen antagonizes manic like behavior in rats: Implications for the development of novel therapeutics for bipolar disorders. Neuropsychology.

[7] Harisson-Read PE (2008). Models of mania and antimanic drug actions: Progressing the endophenotype approach. J Psychophar Macol.

[8] Gimbalvo CT (1992). Protein Kinase C and dopamine transport-2. Effects of amphetamine in Ultro. Neuropharmacology.

[9] Gimbalor CT (1992). Protein Kinase C and dopamine transport-2. Effects of amphetamine in Ultro. Neuropharmacology.

[10] Genegy ME, Hong P, Ferell ST (1993). Phosphorylation of neuromodulin in rat striatum after acute and repeated, intermittent amphetamine. Brain Res Mol Brain Res.

[11] Calabrese B, Halpain S (2005). Essential role for the PKC target MARCKS in maintaining dendritic spine morphology. Neuron.

[12] Jordan VC (1994). Molecular mechanisms of anti-estrogen action in breast cancer. Breast Cancer Res Treat.

[13] R Love R (1992). Effects of tamoxifen on Bone Mineral Density in post-menopausal women with breast cancer. N Engl J Med.

[14] (2010). National Cancer Institute UNIOH, Tamoxifen: Questions and Answers.

[15] Desmaria J (2009). Interaction between tamoxifen and Antidepressants via Cytochrome P4502D6. J Clin Psychiatr.

[16] S Goel S (2009). Effect of Anti-Estrogen on MES and their interaction with Anti-epileptics in Wista- Pats. Pharmacology Online.

[17] Geller B, Craneg JL, Bolhofner K, Nickelsburg MJ, Williams M Phenomenology and Longitudinal course of children with a prepubertal and early adolescent bipolar disorder phenotype Bipolar disorder in childhood and early adolescence. Guilford.

[18] Suppes T, Leverich GS, Keck PE, Nolen WA, Denicoff KD, Zarate CA, Singh JB, Carlsan PJ, Quiroz J, Jolkovsky L, Luckcu Baugh DA (2001). The Stanley foundation bipolar treatment outcome Network Demographics and illness characteristics at the first 261 patients. J Affct Disord.

[19] Smarty S, Find ling RL, Yildiz A, Guleryuz S, Ankerst DP, Ongur D, Renshaw PF (2007). Psychopharmacology of pediatric bipolar disorder: A review. Psychopharmacology.

[20] Del Bello MP, Findling RL (2005). A pilot controlled trial of topiramate for mania in children and adolescents with bipolar disorder. J AM Acad Child Adolesc Psychiatry.

[21] Gabrielle A, Carlson Stephanie E, Meyer C Early onset bipolar disorder. Test book of Psychiatry, Kaplan and Sadock.

[22] Sachs GS, Gardner- Schuster EE (2007). Adjunctive treatment of acute mania: a clinical overview. Acta Psychiatry Scand Suppl.

[23] Fagiolini A, Chengappa KN (2007). Weight gain and metabolic issues of medicines used for bipolar disorder. Curr Psychiatry Rep.

[24] Manji HK, Zarate CA (2002). Molecular and cellular mechanisms underlying mood stabilizations for the development of improved therapeutics. Mol Psychiatry.

[25] Manji HK, Bebchuk JM, Moore Gj, Glitz D, HasanatKa, Chen G (1999). Modulation of CNS signal transduction pathways and gene expression by mood-stabilizing agents. Clin Psychiatry.

[26] Lawrence SE, Faught KA, Vethamat Y, Lawson ML (2004). Beneficial effects of raloxifen and Tamoxifen in the treatment of Pubertal Gynecomastia. J Pediatr.

[27] Bebchuk JM, Artkan CL, Dolan-Manji S, Murphy J, Hasanat K, Manji HK (2000). A preliminary investigation of a protein Kinase C inhibitor in the treatment of acute mania. Arch Gen Psychiatry.

[28] Gould TD, Einat H (2007). Animal models of bipolar disorder and mood stabilize efficacy: a critical head for Improvement. Neurosci Bio Behave Rev.

[29] Ghanizadeh A, Mohammadi MR (2006). Psychometric properties of the Farsi translation of the Kids, Schedule for Affective Disorders and Schizophrenia. BMC Psychiatry.

[30] Young Storm EA (2003). Toward and integration of parent and clinical report on the YMRS. J Affect Disord.

[31] Karimi S (2009). normalization, reliability, validity and analysis of Coax Depression Inventory questionnaire in students in Secondary school, MA thesis in psychology.

[32] Charles F, Armstrong L, Goldman M, Lance L (2013). Drug information Handbook- Tamoxifen.

[33] Carlson GA, Meyer SE (2010). Bipolar disorder Textbook of child and adolescent psychiatry Mina K. Dulcan.

[34] Eugster EA, Rabin SD, Raiter EO (2003). Tamoxifen treatment for precocious puberty in McCune-Albright Syndrom: A multi-center trial. J Pediatr.

[35] Neriss C, Kreher, Erica A (2005). Eugster and R. Ravi Shankar A, Pediatrics.

[36] Lawrence SE, Faught KA, Vethamat Y, Lawson ML (2004). Beneficial effects of raloxifen and Tamoxifen in the treatment of Pubertal Gynecomastia. J Pediatr.

[37] Walter AW, Gajjar A, Reavdon DA (2000). Tamoxifen and cauhoplutin for children with low grade gliomas: A pilot study at St Jude Children’s Research Hospital. J Pediatr Hematol Oncol.

[38] Bebchuk JM, Artkan CL, Dolan-Manji S, Murphy J, Hasanat K, Manji HK (2000). A preliminary investigation of a protein Kinase C inhibitor in the treatment of acute mania. Arch Gen Psychiatry.

[39] Kullcarni J, Garaland KA, Scaffidi A, Headey B, Anderson R, Castella de A (2006). A pilot study of hormone modulation as a new treatment for manic in women with bipolar affective disorder. Psych-neuroendocrinology.

[40] Suppes T, Leverich GS, Keck PE, Nolen WA, Denicoff KD, Zarate CA, Singh JB, Carlsan PJ, Quiroz J, Jolkovsky L, Luckcu Baugh DA (2007). Efficacy of a protein kinase C inhibitor (Tamoxifen) in the treatment of acute mania A pilot study. Bipolar Disorder.

[41] Smarty S, Find ling RL, Yildiz A, Guleryuz S, Ankerst DP, Ongur D, Renshaw PF (2008). Protein Kinase C inhibition in the treatment of manic: a double blind, Placebo controlled of tamoxifen. Arch Gen Psychiatry.

[42] Amrollahi Z, Rezaei F, Salehi B, Modabbernia AH Maroufi A, Esfandiari G (2011). Efficacy and safety of the Tamoxifen adjunctive to lithium in acute bipolar mania. J Affec Disord.

[43] Bebchuk JM (2005). A preliminary investigation of a protein Kinase C inhibitor in the treatment of acute mania. Arch Gen Psychiat.

[44] Kelly C, Lee A (2007). Tamoxifen treatment and New-onset depression in Breast cancer patients. Psychosomatics.

